# Comparison of multidrug-resistant acinetobacter and non-acinetobacter infections in terms of outcome in critically ill patients

**DOI:** 10.1186/2197-425X-3-S1-A129

**Published:** 2015-10-01

**Authors:** A Madaan, V Singh, VJ Singh

**Affiliations:** Patel Super Speciality Hospital, Critical Care Medicine, Jalandhar, India; Sir Ganga Ram Hospital, Critical Care Medicine, New Delhi, India

## Introduction

Acinetobacter infections have increased and gained attention because of the organism's prolonged environmental survival and propensity to develop antimicrobial drug resistance [1],[2]. We performed a retrospective, matched cohort investigation at a tertiary care hospital in a tier II city hospital of India to examine morbidity and mortality outcome of patients with multidrug-resistant (MDR) Acinetobacter (AB) infection compared with patients with MDR non-Acinetobacter (non-AB) infections.

## Objectives

To study the outcome difference in terms of morbidity and mortality in critically ill patients with MDR infections.

## Methods

Patient records from the last 3 years from 2011 through 2013 were studied (*n* = 104) to examine outcomes of hospitalized patient in terms of number of organ dysfunctions, ICU length of stay, hospital length of stay and mortality in patients infected with MDR AB infection compared with the patients with MDR non-AB infection.

## Results

MDR AB-infected patients (*n* = 45, 43%) had greater number oforgan dysfunctions, and higher mean lengths of hospital stay (8.4 days vs. 6.1 days) and ICU stay (5.3 days vs. 0.8 days) after the index day than MDR non-AB infection (*n* = 59, 57%) (Table 1). In-hospital mortality rates for patients with MDR AB infections (42%) were higher than for MDR non-AB infection (10%). *F* critical = 3.934253.

**Table 1:** Table 1

Outcome evaluated	MDR AB	MDR non-AB	p value	f value
Mean length of stay after index day	8.488889	6.101695	0.029299	4.886684
Mean ICU stay after index day	5.311111	0.864407	1.73*10^-11^	57.30083

## Conclusions

We demonstrated in patients infected with MDR strains of any organism that patients infected with Acinetobacter have greater number of organ dysfunction, longer lengths of stay in both the hospital and the ICU as well as increased mortality than patients infected with non-Acinetobacter infection when we controlled for severity of illness.

## References

[1] Mahgoub S (2002). Infection Control Hosp Epidemiol.

[2] Hsueh PR (2002). Emerg Infect Dis.

